# Illumina Sequencing of Common (Short) Ragweed (*Ambrosia artemisiifolia* L.) Reproductive Organs and Leaves

**DOI:** 10.3389/fpls.2016.01506

**Published:** 2016-10-07

**Authors:** Eszter Virág, Géza Hegedűs, Endre Barta, Erzsébet Nagy, Kinga Mátyás, Balázs Kolics, János Taller

**Affiliations:** ^1^Georgikon Faculty, Department of Plant Science and Biotechnology, University of PannoniaKeszthely, Hungary; ^2^Georgikon Faculty, Department of Economic Methodology, University of PannoniaKeszthely, Hungary; ^3^National Agricultural Research and Innovation Centre, Agricultural Biotechnology InstituteGödöllő, Hungary

**Keywords:** Illumina sequencing, transcriptome, common ragweed, male flower, female flower

## Introduction

*Ambrosia artemisiifolia* L. *(A. artemisiifolia, common ragweed)* is one of the most aggressive, rapidly spreading and highly allergenic weeds found in many agricultural settings in the temperate zone. Chemical control of ragweed has some limitation in some crops, therefore it may cause reduced yields both in Europe and North America. The most affected are maize, sunflower, soya bean and pea in Europe (Chollet et al., 1999; Konstantinovic et al., 2005; Chauvel et al., 2006) and grains, tobacco and root crops in North America (Bassett and Crompton, 1975). Pollen allergy of common ragweed affects the human population's quality of life. Its pollen allergens are considered to be major elicitors of type I allergy during late summer and fall inducing respiratory distress such as allergenic rhinitis and seasonal asthma but it is also linked to eczema, ear infections in children and sinusitis (bacterial infection of the sinuses) in adults (Dykewicz, 2003). The spread of this weed therefore causes severe agricultural and public health problems that are important to be solved globally.

For better understanding the genetic regulation of the common ragweed reproduction biology we sequenced the mRNA of flower tissues and leaves of different developmental stages using the Illumina platform. To this end different gender flowers, of this monoecious, dicotyledonous invasive weed were collected from a natural Ambrosia population of a highly infested West-Transdanubian region in Hungary.

The sequence data were assembled de novo to create a reference transcriptome for our future work for this species. Raw reads of the transcriptome assembly have been deposited to NCBI's Sequence Read Archive (SRA) database with the accession numbers SRR3995704 (male flower), SRR3995703 (female flower), and SRR3995705 (leaves). SRA accession: SRP08007. Bioproject ID: PRJNA335689. The Transcriptome Shotgun Assembly project has been deposited at DDBJ/ENA/GenBank under the accession GEZL00000000. The version described in this paper is the first version, GEZL01000000.

The presented data were used for the first time to determine the complete coding sequence and putative signal peptide of an Amb a 3 allergen isoform of *A. artemisiifolia* recently (Taller et al., 2016).

## Materials and methods

### Plant materials

Three sample types, such as male (♂) and female (♀) flowers from initial to final developmental stages and leaves of different developmental stage and position of *A. artemisiifolia* plants were collected from beside an agricultural field in West-Transdanubian region of Hungary (46⋅ 44′ 55.4″ N, 17⋅ 14′ 20.1″ E) during the whole flowering period from middle of July to the end of August. Samples were frozen immediately in liquid nitrogen and stored at −80⋅C until further use. The highly pure total RNA from plant tissues were isolated using TaKaRa Plant RNA Extraction Kit according to manufacturer's instructions (Takara Bio Inc; Japan). Purity and concentration of all RNA samples were quantified spectrophotometrically using Agilent 2100 Bioanalyzer (Agilent Technologies; USA).

### Enrichment of mRNA, cDNA synthesis and library preparation for Illumina HiSeq paired-end sequencing

For poly-A based mRNA enrichment and cDNA synthesis the Illumina TruSeq™ RNA sample preparation kit (Low-Throughput protocol) was used according to manufacturer's instructions. Briefly, 1.5 μg of total RNA sample of male, female and leaf tissues were used for poly-A mRNA selection using streptavidin-coated magnetic beads. Two rounds of enrichment for poly-A mRNA was performed followed by thermal mRNA fragmentation. The cDNA was synthesized from enriched and chemically fragmented RNA using reverse transcriptase (Super-Script II) and random primers. cDNA was converted into double stranded (ds) DNA using the reagents supplied in the kit and ~0.5–1 μg from each sample were used for the library preparation. The RNA-Seq was performed using Illumina HiSeq2000 system. The hybridization onto a flow cell, the dsDNA fragments were blunt-ended through an end-repair reaction and both ends were ligated to platform-specific double-stranded bar-coded adapters. The samples were run in one lane using multiple indexing adapters. For library amplification an adapter-selective PCR reaction was performed. In order to avoid the skewing of the library representation the number of PCR cycles was minimized to 15. The optimum cluster density of libraries was created by qPCR according to *qPCR Quantification Protocol Guide*. The size and purity of the samples were checked by Agilent 2100 Bioanalyzer (Agilent Technologies, USA). The PCR products were at 260 bp, approximately. The DNA libraries were multiplexed normalizing them to 10 nM.

### *De novo* assembling and analysis of high throughput sequencing data

Quality control and preprocessing of the 2 × 100 bp raw reads was done with FastQC (Andrews, 2010) and are summarized in Table 1. After quality trimming at a Phred score ≥ 28 a de novo assembly of the combined three transcriptome sequencing datasets was performed using Trinity (Haas et al., 2013) with 25 k-mer size. The resulted contigs of the assembly were used later as the reference transcriptome of the common ragweed. The statistics of the assembly are summarized in Table 2. The reads from the samples were then mapped separately back to reference using the short read aligner program Bowtie (Langmead et al., 2009). The transcript abundances were estimated using the RSEM program (http://deweylab.github.io/RSEM/) with the scripts provided in the Trinity package. A collection of 162494 unigenes (average length of 391 bp) was generated and their annotation was performed using Trinotate suit (https://trinotate.github.io/). We found that 72.46 and 68,4 % of the unigenes had significant similarity with proteins in the National Center for Biotechnology Information (NCBI) non-redundant protein database (Nr) and Swiss-Prot database respectively.

**Table 1:** **Quality control of raw reads obtained by HiSeq2000**.

**Sample**	**Total raw reads**	**Read length**	**Average Phred score**	**GC%**
♂ flower	24110256	100	36	52
♀ flower	23264636	100	36	54
leaf	24330693	100	37	46

**Table 2:** **Statistics of transcriptome assembling**.

**Attributes**	**Value**
Total trinity genes	162494
Total trinity transcripts	229116
GC%	41.61
Contig N50	774
Median contig length	391
Total assembled reads	1377646

## Direct link to deposited data and information to users

The raw reads are available in Fastq format at the following link http://www.ncbi.nlm.nih.gov/sra/SRP080078.

The transcriptome shotgun assembly is available in FASTA format at the following link http://www.ncbi.nlm.nih.gov/nuccore/GEZL00000000.

Users can download and use the data freely for research purpose only with quoting this paper as reference to the data.

## Author contributions

EV: wrote the manuscript, performed cDNA library preparation, bioinformatics and analysis on the data; EB: performed transcriptome assembling and bioinformatics; GH: performed statistical analysis and informatics operations; EN, BK, and KM: collected the samples and performed RNA isolation; JT: supervised the project and acquired funding.

### Conflict of interest statement

The authors declare that the research was conducted in the absence of any commercial or financial relationships that could be construed as a potential conflict of interest.
